# Expression and Cleavage of Middle East Respiratory Syndrome Coronavirus nsp3-4 Polyprotein Induce the Formation of Double-Membrane Vesicles That Mimic Those Associated with Coronaviral RNA Replication

**DOI:** 10.1128/mBio.01658-17

**Published:** 2017-11-21

**Authors:** Diede Oudshoorn, Kevin Rijs, Ronald W. A. L. Limpens, Kevin Groen, Abraham J. Koster, Eric J. Snijder, Marjolein Kikkert, Montserrat Bárcena

**Affiliations:** a Molecular Virology Laboratory, Department of Medical Microbiology, Leiden University Medical Center, Leiden, The Netherlands; b Section Electron Microscopy, Department of Molecular Cell Biology, Leiden University Medical Center, Leiden, The Netherlands; Vanderbilt University Medical Center

**Keywords:** convoluted membranes, electron tomography, membrane structure, nidoviruses, nonstructural proteins, replication complex, replication organelle biogenesis, replication structures, viral factory, viral protein

## Abstract

Betacoronaviruses, such as Middle East respiratory syndrome coronavirus (MERS-CoV), are important pathogens causing potentially lethal infections in humans and animals. Coronavirus RNA synthesis is thought to be associated with replication organelles (ROs) consisting of modified endoplasmic reticulum (ER) membranes. These are transformed into double-membrane vesicles (DMVs) containing viral double-stranded RNA and into other membranous elements such as convoluted membranes, together forming a reticulovesicular network. Previous evidence suggested that the nonstructural proteins (nsp’s) 3, 4, and 6 of the severe acute respiratory syndrome coronavirus (SARS-CoV), which contain transmembrane domains, would all be required for DMV formation. We have now expressed MERS-CoV replicase self-cleaving polyprotein fragments encompassing nsp3-4 or nsp3-6, as well as coexpressed nsp3 and nsp4 of either MERS-CoV or SARS-CoV, to characterize the membrane structures induced. Using electron tomography, we demonstrate that for both MERS-CoV and SARS-CoV coexpression of nsp3 and nsp4 is required and sufficient to induce DMVs. Coexpression of MERS-CoV nsp3 and nsp4 either as individual proteins or as a self-cleaving nsp3-4 precursor resulted in very similar DMVs, and in both setups we observed proliferation of zippered ER that appeared to wrap into nascent DMVs. Moreover, when inactivating nsp3-4 polyprotein cleavage by mutagenesis, we established that cleavage of the nsp3/nsp4 junction is essential for MERS-CoV DMV formation. Addition of the third MERS-CoV transmembrane protein, nsp6, did not noticeably affect DMV formation. These findings provide important insight into the biogenesis of coronavirus DMVs, establish strong similarities with other nidoviruses (specifically, the arteriviruses), and highlight possible general principles in viral DMV formation.

## IMPORTANCE

The RNA replication of positive stranded RNA viruses of eukaryotes is thought to take place at cytoplasmic membranous replication organelles (ROs). Double-membrane vesicles are a prominent type of viral ROs. They are induced by coronaviruses, such as SARS-CoV and MERS-CoV, as well as by a number of other important pathogens, yet little is known about their biogenesis. In this study, we explored the viral protein requirements for the formation of MERS-CoV- and SARS-CoV-induced DMVs and established that coexpression of two of the three transmembrane subunits of the coronavirus replicase polyprotein, nonstructural proteins (nsp’s) 3 and 4, is required and sufficient to induce DMV formation. Moreover, release of nsp3 and nsp4 from the polyprotein by proteolytic maturation is essential for this process. These findings provide a strong basis for further research on the biogenesis and functionality of coronavirus ROs and may point to more general principles of viral DMV formation.

## INTRODUCTION

**FIG 1:**
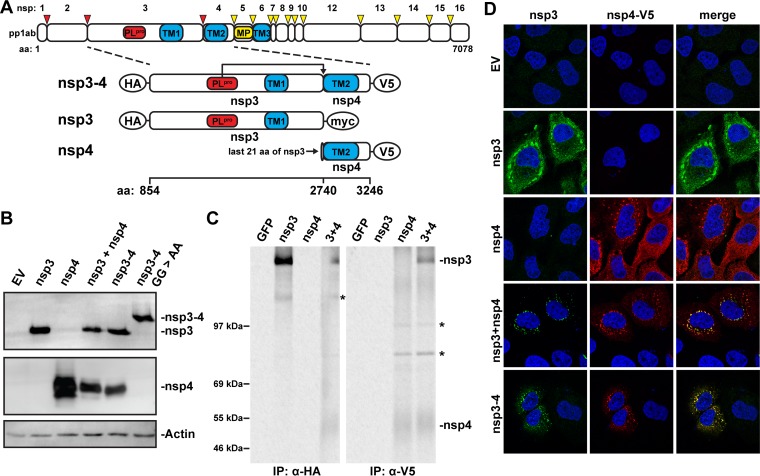
MERS-CoV nsp3 and nsp4 interact with each other. (A) Scaled schematic overview of MERS-CoV pp1ab and nsp3-4 constructs. Amino acid numbers refer to the MERS-CoV pp1ab sequence. The expected cleavage of the nsp3/nsp4 junction by PL^pro^ is indicated. The epitope tags used at the termini of the constructs are indicated with ovals. TM, transmembrane region. (B) 293T cells were transfected with MERS-CoV nsp3-4 plasmids or empty pCAGGS vector (EV) and analyzed by Western blotting 20 h posttransfection. nsp3 was detected with anti-SARS-CoV nsp3 serum that cross-reacts with MERS-CoV nsp3 (21), and nsp4 was detected with anti-V5 monoclonal antibody. (C) Constructs expressing MERS-CoV nsp3 or nsp4 or a GFP control were transfected into 293T cells, which were metabolically labeled with [^35^S]methionine-cysteine from 4 to 20 h posttransfection. Lysates were immunoprecipitated with the indicated antibodies, separated on an SDS-PAGE gel, and visualized using phosphorimaging. Bands not corresponding to expected protein size in the Western blot are indicated with asterisks. The ~130-kDa band in the nsp3 IP was also observed in the Western blot. nsp4 bands in IP were fuzzy likely due to the relatively high hydrophobicity of the protein. (D) HuH-7 cells were transfected with the indicated plasmids, and localization of MERS-CoV nsp3 and nsp4 was analyzed using immunofluorescence labeling and confocal microscopy at 24 h posttransfection. nsp3 was detected with anti-SARS-CoV-nsp3 serum, and nsp4 was detected with anti-V5 monoclonal antibody.

Coronaviruses are positive stranded RNA viruses that can pose serious zoonotic threats to human health, as evidenced by the emergence of severe acute respiratory syndrome coronavirus (SARS-CoV) in 2002 (1, 2) and, more recently, the Middle East respiratory syndrome coronavirus (MERS-CoV). Since the start of the outbreak in 2012, MERS-CoV has continued to circulate in the Arabian Peninsula (3, 4), which to date has led to over 2,000 laboratory-confirmed human infections with a lethality rate of about 35% (http://www.who.int/emergencies/mers-cov/en/). Coronaviruses, members of the order *Nidovirales*, have the largest known positive stranded RNA genomes, ranging from 26 to 33.5 kb (5–7). The 5′-proximal two-thirds of the genome contains the replicase gene that consists of two open reading frames (ORF1a and ORF1b). ORF1a translation yields polyprotein 1a (pp1a; roughly 4,000 to 4,500 amino acid [aa] residues long), which, following a −1 ribosomal frameshift, can be extended with the ORF1b-encoded polyprotein to yield pp1ab (6,700 to 7,200 aa residues in total). The pp1a and pp1ab polyproteins contain the enzymes of the RNA-synthesizing complex that drives viral genome replication and subgenomic mRNA synthesis (8). The replicase polyproteins are co- and post-translationally processed into 15 or 16 nonstructural proteins (nsp’s) by two or three ORF1a-encoded proteases (9–13). Depending on the coronavirus, one or two papain-like proteases (PL^pro^) that reside in nsp3 process the part of the polyproteins upstream of nsp4. In all coronaviruses, the region downstream of nsp4 is cleaved by the 3C-like cysteine protease or main protease (M^pro^) located in nsp5 (Fig. 1A) (9–13).

Coronaviruses, like all positive stranded RNA viruses of eukaryotes, hijack intracellular membranes to form their replication organelles (ROs) (14–18). These generally reside in the perinuclear region of the cell and are assumed to constitute microenvironments that promote viral RNA synthesis while possibly shielding replicative intermediates, specifically double-stranded RNA, from detection by the innate immune system. The most prominent membrane structures induced after coronavirus infection are double-membrane vesicles (DMVs) (19–28), which appear to contain double-stranded RNA, a frequently used marker of positive stranded RNA virus replication (19, 29). DMVs are not only formed during the replication of coronaviruses but are also a central component of the ROs induced by several other plus-stranded RNA viruses such as hepatitis C virus (HCV) and enteroviruses like poliovirus and coxsackievirus (30–32). Most of our current knowledge of coronavirus ROs has been gained through electron microscopy (EM) studies of members of the genus *Betacoronavirus*, which include SARS-CoV, MERS-CoV, and mouse hepatitis virus (MHV) (19–24). Electron tomography (ET) studies of SARS-CoV-infected cells showed that DMV outer membranes are often interconnected and also connect to the endoplasmic reticulum (ER) and/or another virus-induced structure called convoluted membranes (CM) (19). Together, they form an elaborate reticulovesicular network (RVN), for which the ER probably serves as the membrane donor (19).

Given their membrane-spanning features, the nsp3, nsp4, and nsp6 subunits of the coronavirus replicase are the prime candidates for directing RO formation (Fig. 1A) (17, 33). Each of these proteins spans the membrane multiple times (2, 4, and 6 times, respectively), and they have 1, 2, and 3 luminal loops, respectively, with both nsp3 and nsp4 having a large luminal domain (34–37). Mutagenesis studies showed that the first luminal loop of MHV nsp4 is critical for viral replication (38, 39). Furthermore, nsp4s of both MHV and SARS-CoV contain sites (2 and 1, respectively) for N-linked glycosylation in the first luminal loop of nsp4 (34, 37, 40). When both these sites were mutated in MHV nsp4 (38, 40), the virus was attenuated in cell culture and DMV formation was impaired, suggesting that nsp4 plays a critical role in coronaviral RO formation. The combined membrane-spanning regions of these proteins (i.e., including all luminal loops and flanking transmembrane domains) are commonly referred to as TM1, TM2, and TM3, respectively. nsp3, nsp4, and nsp6 are nonconventional transmembrane proteins in the sense that they are derived from a polyprotein and do not contain N-terminal signal sequences for cotranslational membrane insertion. It is currently unknown how their membrane insertion is accomplished and whether polyprotein cleavage precedes (or is perhaps required for) translocation across the ER membrane. To a certain extent, nsp2, nsp3, and nsp5 of the distantly related arteriviruses (also members of the order *Nidovirales*) can be considered equivalent to coronavirus nsp3, nsp4, and nsp6, in terms of their relative position in the replicase polyprotein and their membrane-spanning properties. For arteriviruses, expression of nsp2 and nsp3 alone was necessary and sufficient for the formation of double-membrane structures strikingly resembling the DMVs observed in infected cells (41). Coexpression of nsp5 reduced the size of the induced DMVs but did not change their overall architecture (18). In the case of coronaviruses, it was recently reported that the transient coexpression of SARS-CoV nsp3, nsp4, and nsp6 led to the formation of DMVs (42). Cells coexpressing nsp3 and nsp4 alone contained so-called maze-like bodies (MLBs), consisting of paired ER membranes (zippered ER) and some circular profiles that were interpreted as cross sections of double-membrane tubules. Therefore, it was concluded that nsp6 is essential for the biogenesis of SARS-CoV DMVs, whereas nsp3 and nsp4 can mediate the pairing of membranes that are likely an intermediate in DMV formation (42).

In the current study, we examined the role of MERS-CoV nsp’s in betacoronavirus RO biogenesis. Using EM and ET, we found that MERS-CoV nsp3 and nsp4, either coexpressed from separate plasmids or expressed as a self-cleaving polyprotein fragment (nsp3-4), are essential and sufficient for the formation of DMVs that assemble into an RVN. Addition of the third transmembrane subunit of the MERS-CoV replicase, nsp6, did not alter the overall morphology of the induced DMVs. When nsp3-4 polyprotein processing was prevented by mutagenesis, this blocked the formation of DMVs while membrane pairing did still occur, strongly suggesting that proteolytic processing coordinates DMV formation in time and/or space. To compare our results for MERS-CoV with the previous work on SARS-CoV (42), we used ET to analyze the three-dimensional (3D) structure of the maze-like bodies induced upon coexpression of SARS-CoV nsp3 and nsp4 and were thus able to conclude that the circular profiles observed in that setting in fact correspond to DMVs rather than tubules. This established that, also in the case of SARS-CoV, coexpression of nsp3 and nsp4 suffices to induce DMV formation. Together, our results provide important new insights regarding the biogenesis of coronavirus ROs and demonstrate the conservation of certain principles underlying RO formation, both among the coronaviruses and in comparison to more distantly related members of the order *Nidovirales*.

## RESULTS

### MERS-CoV nsp3 and nsp4 colocalize in the perinuclear region of the cell.

To study whether the transmembrane nsp’s of MERS-CoV are able to induce DMV formation, we expressed nsp3 and nsp4 from a CAG promoter (43) either by cotransfection of cells with plasmids encoding individual proteins or by transfection with a single plasmid encoding a self-cleaving nsp3-4 polyprotein fragment (Fig. 1A; Table S1). Constructs were codon optimized for expression in human cells, potential splice sites were eliminated, and the encoded proteins were equipped with hemagglutinin (HA), myc, or V5 tags at their termini. The constructs were transfected into 293T cells to verify protein expression and processing (Fig. 1B). The wild-type nsp3-4 polyprotein was fully cleaved into mature nsp3 and nsp4, as was previously described (44). As a control, a mutant in which the nsp3/nsp4 cleavage site was inactivated (G2739A/G2740A; GG>AA) (45) was included to generate the noncleaved precursor. Interactions between nsp3 and nsp4 were previously shown to occur for MHV and SARS-CoV (46, 47), and we assessed whether this was also the case for the corresponding MERS-CoV proteins. To this end, 293T cells were transfected with a construct expressing HA-nsp3-myc or nsp4-V5 or cotransfected with both constructs. Expression products were labeled metabolically with [^35^S]methionine and [^35^S]cysteine and subsequently immunoprecipitated with either HA- or V5-specific antibodies (Fig. 1C). Upon immunoprecipitation with the HA-specific antiserum, nsp4-V5 was brought down when HA-nsp3 was present (left panel). Conversely, when using the V5-specific antibody, HA-nsp3 was coimmunoprecipitated when nsp4-V5 was present (right panel). These findings demonstrated that these two MERS-CoV proteins interact and further supported the notion that this is a common feature of coronaviruses.

10.1128/mBio.01658-17.3 TABLE S1  Details of MERS-CoV expression constructs. The parts of MERS-CoV pp1a used for the different expression constructs are listed in the table. Amino acid numbers are based on pp1a of the MERS-CoV EMC/2012 strain. The coding sequence was human codon optimized unless otherwise indicated. Download TABLE S1, XLSX file, 0.01 MB. Copyright © 2017 Oudshoorn et al. 2017 Oudshoorn et al. This content is distributed under the terms of the Creative Commons Attribution 4.0 International license.

When using immunofluorescence microscopy, separate expression of nsp3 or nsp4 in HuH-7 cells yielded a reticular labeling pattern, with some more-intense foci in the perinuclear region of the cell, suggesting that—in the absence of the other—either protein localized at least partially to the ER (Fig. 1D). This reticular pattern (but without the foci) has been described previously upon transient expression of MHV and SARS-CoV nsp4 (34, 48), whereas full-length SARS-CoV nsp3 was reported to localize to foci similar to those that we observed (42). When coexpressing MERS-CoV nsp3 and nsp4 or when expressing the self-cleaving nsp3-4 polyprotein, the reticular pattern was much less pronounced and the two proteins mainly colocalized in foci in the perinuclear region (Fig. 1D, lower panels). This was in agreement with the finding that MERS-CoV nsp3 and nsp4 interact and suggested that this interaction strongly promotes their recruitment to the foci in the perinuclear region.

### MERS-CoV nsp3 and nsp4 are required and sufficient to induce DMV formation.

**FIG 2:**
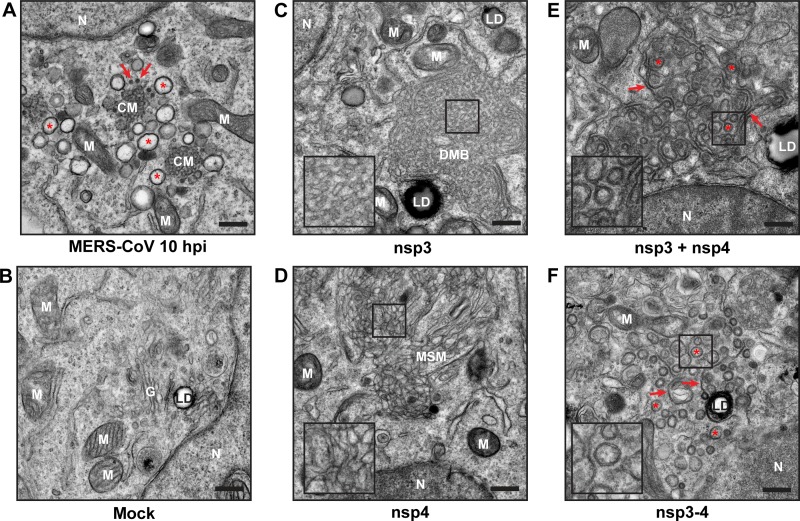
MERS-CoV nsp3 and nsp4 induce modification of intracellular membranes. (A and B) HuH-7 cells were infected with MERS-CoV (A) or mock infected (B) and analyzed at 10 h p.i. using EM. Several DMVs are indicated with red asterisks, and several spherules are indicated with red arrows. (C to F) HuH-7 cells were transfected with constructs expressing either individual nsp’s (C and D) or both nsp3 and nsp4, following either cotransfection with two plasmids (nsp3 + nsp4) or expression of a self-cleaving precursor (nsp3-4) (E and F), and analyzed using EM at 24 h posttransfection. (E and F) Some stretches of zippered ER are indicated with red arrows, and several DMVs are indicated with red asterisks. N, nucleus; G, Golgi apparatus; M, mitochondria; LD, lipid droplet; CM, convoluted membranes; DMB, disordered-membrane body; MSM, clusters of modified single membranes. Bars, 500 nm.

The next step was to determine whether nsp3 and nsp4 could induce the formation of double-membrane structures similar to those observed during infection. As a reference, MERS-CoV-infected HuH-7 cells were analyzed by EM. The membrane structures that were previously described in high-pressure frozen and freeze-substituted Vero cells infected with MERS-CoV (21) were readily apparent at 10 h postinfection (p.i.) in chemically fixed HuH-7 cells (Fig. 2A). Numerous DMVs were found (red asterisks), often adjacent to areas containing CM. The DMV interior appeared electron translucent, a difference from cryofixed samples (21) that can likely be attributed to the different sample preparation method, as the contents of CoV-induced DMVs are easily lost upon chemical fixation (22, 24, 28). Occasionally some smaller circular profiles were observed that seemed similar in size to the spherules recently described for the gammacoronavirus infectious bronchitis virus (IBV) (red arrows) (28). None of these structures was found in mock-infected control samples (Fig. 2B).

When HuH-7 cells expressed either nsp3 or nsp4, areas containing modified membranes were observed, which likely corresponded to the foci observed in fluorescence microscopy (Fig. 1D). In nsp3-expressing cells (Fig. 2C), we detected large regions, usually several micrometers in diameter, of disordered membrane bodies (DMBs), which were similar to those previously observed after SARS-CoV nsp3 expression (42). The membrane structures clustering in these DMBs were reminiscent of the surrounding ER cisternae, with which they were frequently connected, suggesting that DMBs consisted of clustered ER-derived membranes. Upon expression of MERS-CoV nsp4, large clusters of modified single membranes (MSM) were observed (Fig. 2D), but these structures seemed more irregular than those induced by nsp3 (Fig. 2C). The expression of SARS-CoV nsp4 did not result in changes in intracellular membrane morphology (42), in contrast with our present observations following MERS-CoV nsp4 expression. Whether this reflects differences between the experimental setups used or an actual difference between these viral proteins remains to be determined.

When MERS-CoV nsp3 and nsp4 were expressed in the same cell, either by cotransfection or by expression of the self-cleaving nsp3-4 polyprotein, a remarkably different set of membrane structures was observed (Fig. 2E and F). A combination of circular double-membrane profiles (red asterisks) and paired membranes (red arrows) was present in both cases, suggesting that the combined expression of MERS-CoV nsp3 and nsp4 is sufficient to induce DMV formation. There was no apparent difference between the structures resulting from coexpression of nsp3 and nsp4 and those resulting from expression of the self-cleaving nsp3-4 polyprotein (Fig. 2E and F), but in both cases the circular profiles were significantly smaller than the ones observed in MERS-CoV-infected cells (average diameters of 146 and 148 nm, respectively, versus 252 nm in infection) (see Fig. S1 in the supplemental material).

10.1128/mBio.01658-17.1 FIG S1  Structures induced by MERS-CoV nsp3 and nsp4 are smaller than those observed during MERS-CoV infection. The size of circular profiles was determined for MERS-CoV-infected cells (10 h p.i.), as well as for cells expressing nsp3 and nsp4 (cotransfected), the nsp3-4 self-cleaving precursor, and the nsp3-5-GFP-6 construct. One hundred profiles were measured for each condition and plotted as a box-and-whisker plot. The boxes show the 25th to 75th percentile, and the whiskers show the total range of the measurements. Download FIG S1, TIF file, 0.3 MB. Copyright © 2017 Oudshoorn et al. 2017 Oudshoorn et al. This content is distributed under the terms of the Creative Commons Attribution 4.0 International license.

These membrane modifications were frequently found in all the samples analyzed. In order to further investigate how the frequency of EM-positive cells compared to the transfection efficiency, a quantitative analysis was carried out on samples of cells expressing MERS-CoV nsp3-4. Immunofluorescence microscopy showed that approximately 40% of the transfected cells were positive for expression of MERS-CoV nsp3 and nsp4 (*n* = 174), while around 19% of the cell sections (*n* = 288) contained double-membrane structures. Both DMVs and zippered ER clustered together in all the EM positive cell sections, although at slightly different ratios (see Fig. S2 for a gallery). As the EM analysis was based on one random section per cell (~100 nm thick) that may not always capture the region with membrane modifications, it was not surprising that the fraction of positive cells observed in EM was smaller than that observed in whole cells using light microscopy. The numbers above in fact strongly suggest that the formation of DMVs and zippered ER is induced in most, if not all, cells expressing MERS-CoV nsp3 and nsp4.

10.1128/mBio.01658-17.2 FIG S2  Gallery of double-membrane structures induced by expression of MERS-CoV nsp3-4. HuH-7 cells were transfected with plasmids encoding MERS-CoV nsp3-4. The images display areas with double-membrane structures in different cells and give an overview of the variability encountered in the EM analysis (*n* = 288 cell sections). Putative DMVs (circular profiles) and zippered ER clustered together in the cells, although at slightly different ratios. In each image, two of the visible putative DMVs are marked with red asterisks, while the red arrows point at zippered ER stretches. N, nucleus; M, mitochondria; LD, lipid droplet. Bars, 500 nm. Download FIG S2, TIF file, 3.3 MB. Copyright © 2017 Oudshoorn et al. 2017 Oudshoorn et al. This content is distributed under the terms of the Creative Commons Attribution 4.0 International license.

**FIG 3:**
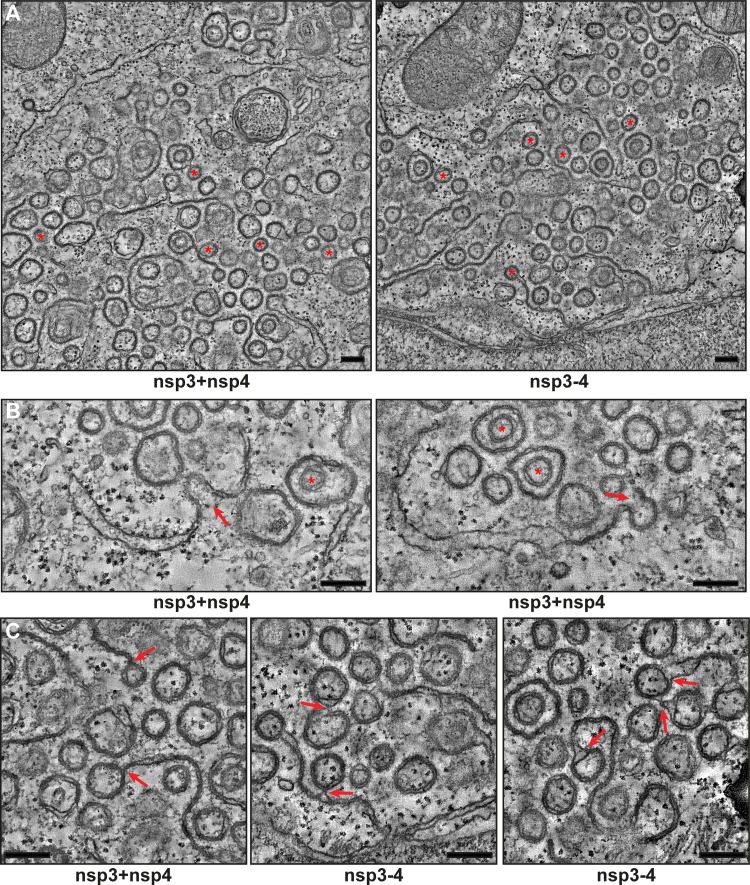
MERS-CoV nsp3 and nsp4 induce the formation of DMVs that are organized in an RVN. HuH-7 cells were cotransfected with constructs expressing nsp3 and nsp4 or the nsp3-4 precursor and fixed for ET analysis. (A) Overviews of reconstructed tomograms (available as Movies S1 and S2, respectively) for both conditions. Some of the fully reconstructed closed DMVs are indicated with red asterisks. (B) Zippered ER curving into putative intermediates during DMV biogenesis (indicated with red arrows) is shown. Two DMVs that are enclosed within other DMVs are indicated with red asterisks. (C) Examples of connections between DMVs and (zippered) ER (indicated with red arrows). All the images are virtual 5-nm-thick slices from the reconstructed tomograms. Bars, 250 nm.

While the circular profiles observed were suggestive of double-membrane vesicle formation, they could also correspond to cross sections of double-membrane tubular structures. To resolve this issue, we obtained 3D reconstructions of these membrane structures using ET (Fig. 3; Movies S1 and S2), which confirmed that genuine DMVs were indeed formed upon expression of either nsp3 plus nsp4 or nsp3-4 of MERS-CoV. The distinctive feature that unambiguously identifies a vesicle in a tomogram is a circular profile that is largest at the vesicle’s equator and decreases in diameter when moving up or down from that plane through successive tomographic slices until, if the vesicle is fully contained in the section, it disappears. Indeed, many profiles like this were observed in the tomograms (Fig. 3A, red asterisks; Movies S1 and S2, green dots). We found no openings connecting the DMV interior and the cytosol, similar to what was observed previously upon tomographic analysis of coronavirus-infected cells (19, 28).

10.1128/mBio.01658-17.4 MOVIE S1  Three-dimensional reconstruction of the membrane structures induced by coexpression of MERS-CoV nsp3 and nsp4 as individual subunits. Tomogram of the region shown in Fig. 3A (left), which was observed upon cotransfection of HuH-7 cells with constructs expressing MERS-CoV nsp3 and nsp4. The movie shows consecutive virtual slices (1.67 nm thick) throughout the tomogram. Some of the DMVs that were fully reconstructed (i.e., originally contained in the cell section) are indicated with green dots. Bar, 500 nm. Download MOVIE S1, AVI file, 18.7 MB. Copyright © 2017 Oudshoorn et al. 2017 Oudshoorn et al. This content is distributed under the terms of the Creative Commons Attribution 4.0 International license.

10.1128/mBio.01658-17.5 MOVIE S2  Three-dimensional reconstruction of the membrane structures induced by coexpression of MERS-CoV nsp3 and nsp4 as a self-cleaving precursor. Tomogram of the region shown in Fig. 3A (right) observed upon transfection of HuH-7 cells with a construct expressing the MERS-CoV nsp3-4 precursor. The movie shows consecutive virtual slices (1.67 nm thick) throughout the tomogram. Some of the DMVs that were fully reconstructed (i.e., originally contained in the cell section) are indicated with green dots. Bar, 500 nm. Download MOVIE S2, AVI file, 19.5 MB. Copyright © 2017 Oudshoorn et al. 2017 Oudshoorn et al. This content is distributed under the terms of the Creative Commons Attribution 4.0 International license.

The tomograms corroborated the structural similarity between the membrane structures induced by cotransfection with nsp3 and nsp4 constructs and by expression of the self-cleaving nsp3-4 polyprotein. The electron density of the DMV interior seemed similar to that of the surrounding cytoplasm, and in this sense, it was different from that of DMVs in MERS-CoV-infected cells (compare to Fig. 3A), which is likely due to the absence of other viral proteins and double-stranded RNA. In some cases, DMVs appeared to be contained in a larger double-membrane structure (Fig. 3B, red asterisks). Such structures have not been observed in coronavirus-infected cells. The paired membranes were often continuous with ER cisternae (Fig. 3B) and resembled the so-called zippered ER that has also been observed in IBV-infected cells (28), although they have not been documented so far for betacoronavirus-infected cells. These paired membranes may represent an intermediate of DMV biogenesis. Further supporting this explanation, structures in which the zippered ER seemed to transform into a nascent DMV could readily be observed in the tomograms (Fig. 3B, red arrows). We also observed DMV-DMV, DMV-zippered ER, and DMV-ER connections (Fig. 3C, red arrows), whereas completely isolated DMVs were in fact rare.

In summary, while the described differences between nsp3-4-expressing and MERS-CoV-infected cells suggest that other viral components may modulate the process of DMV formation and would be required to form the full array of membrane structures observed during infection, our results establish that MERS-CoV nsp3 and nsp4 are sufficient to trigger all the membrane-remodeling steps required for inducing DMV formation, likely through the transformation of ER membranes into an RVN consisting of DMVs and modified ER.

### MERS-CoV nsp6 does not alter DMV morphology.

The DMVs induced by expression of MERS-CoV nsp3 and nsp4 largely mimicked those observed during infection. However, the additional RVN elements that have been observed in this and previous studies of coronavirus-infected cells (CM and spherules) were not detected. To investigate whether nsp6, the third transmembrane subunit of the coronavirus replicase, plays a role in their formation or affects DMV formation, we aimed to extend the expressed polyprotein fragment to include nsp5 and nsp6. In addition to PL^pro^ cleaving the nsp3/nsp4 site, this should lead to processing of the nsp4/nsp5 and nsp5/nsp6 junctions by the nsp5-based M^pro^, an assumption based on sequence conservation and studies performed with other coronaviruses (3), as the kinetics of MERS-CoV polyprotein processing in cell-based assays have not been documented in any detail.

**FIG 4:**
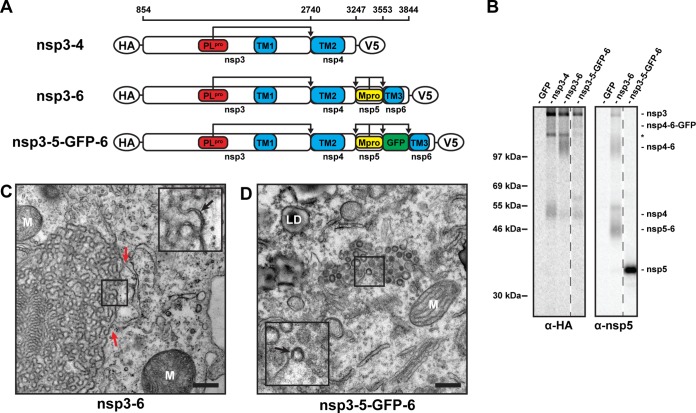
Coexpression of MERS-CoV nsp6 does not alter DMV morphology. (A) Scaled schematic overview of MERS-CoV nsp3-6 constructs. Amino acid numbers at the top are the positions in MERS-CoV pp1a. Expected sites of cleavage by PL^pro^ and M^pro^ are indicated. TM, transmembrane domain. Epitope tags used are indicated with ovals. (B) 293T cells were transfected with indicated plasmids and metabolically labeled with [^35^S]methionine-cysteine from 4 to 20 h posttransfection. Lysates were immunoprecipitated with indicated antibodies, separated on an SDS-PAGE gel, and imaged using a phosphorimager. The ~130-kDa band in samples precipitated with anti-HA serum (indicated with an asterisk) is the same as the one observed when only nsp3 was expressed (Fig. 1C). nsp4 bands and putative nsp4- and nsp6-containing precursor bands were fuzzy, likely due to their relatively large hydrophobic domains. (C and D) HuH-7 cells were transfected with indicated plasmids and analyzed using EM at 24 h posttransfection. Red arrows indicate possible connections between the ER and the cubic membranes. The insets show some areas where double membranes can be observed. M, mitochondria; LD, lipid droplet. Bars, 500 nm.

Remarkably, however, when the “regular” nsp3-6 polyprotein was expressed, efficient processing of the nsp3/4 site was achieved, but nsp’s located downstream of this junction were retained in processing intermediates due to poor cleavage of the nsp4/5 and nsp5/6 junctions, as observed by immunoprecipitation (IP) analysis (Fig. 4A and B). This prompted us to design a set of alternative polyprotein constructs to investigate and optimize the proteolytic autoprocessing of the nsp3-6 region (see Table S1). Efficient cleavage at all sites was observed only for an engineered polyprotein (nsp3-5-GFP-6) in which green fluorescent protein (GFP) had been inserted between two copies of the nsp5/6 cleavage site (Fig. 4A). Immunoprecipitation analysis established that this nsp3-5-GFP-6 polyprotein was processed into four separate nsp’s and GFP (Fig. 4B). Consequently, this construct could be used to evaluate the effect of expressing nsp6 in addition to nsp3 and nsp4.

When HuH-7 cells expressed the “regular” nsp3-6 polyprotein, which was barely cleaved at the nsp5/6 junction (Fig. 4B), we no longer detected the DMVs previously observed upon nsp3-4 expression. Instead, large areas of highly organized and curved membrane structures were seen (Fig. 4C), which were connected to surrounding ER cisternae (Fig. 4C, red arrows). In contrast to the large single-membrane clusters observed in nsp3- or nsp4-expressing cells (Fig. 2C and D), they consisted of double membranes (Fig. 4C, black arrow in the inset). The geometric pattern in these large areas containing double-membrane structures is typical of cubic membranes (49), which can result from overexpression and/or misfolding of ER proteins, leading to protein and membrane aggregation. In contrast, when HuH-7 cells expressed the engineered nsp3-5-GFP-6 polyprotein, which was almost fully processed (see above), cubic membranes were not observed and we found instead putative DMVs together with zippered ER (Fig. 4D), structures very similar to the ones found in cells expressing just nsp3 and nsp4 (compare with Fig. 2E and F). Also, the average size of these DMVs (146 nm) was comparable to that of DMVs induced by nsp3-4 expression (148 nm) (Fig. S1). Circular profiles (putative DMVs) were detected in 33 out of 642 cell sections analyzed; however, none of these regions contained CM or spherules. This suggests that, while nsp3 and nsp4 are necessary and sufficient to induce the rearrangement of intracellular membranes into DMVs, the presence of (cleaved) nsp6 does not suffice to trigger the formation of the additional membrane structures typical of MERS-CoV infection. Other viral components that are present during MERS-CoV replication, such as viral RNA or other viral proteins, might thus be required for the formation of convoluted membranes and spherules.

### Cleavage of the MERS-CoV nsp3/nsp4 junction is essential for DMV formation.

**FIG 5:**
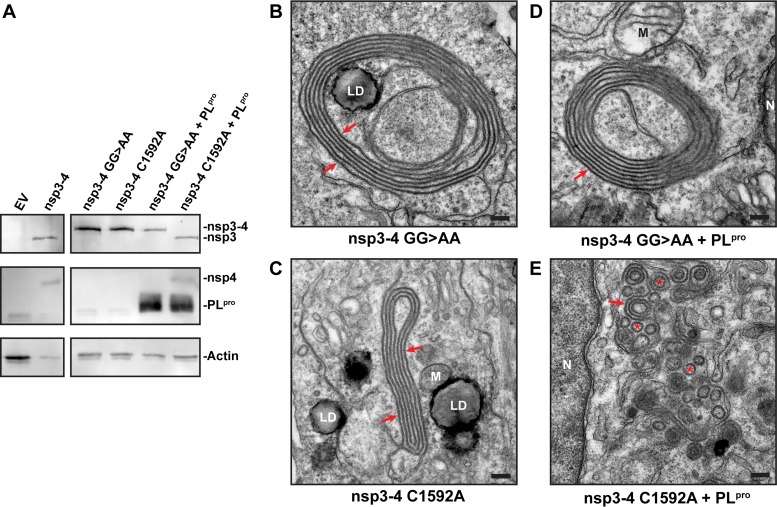
Cleavage of MERS-CoV nsp3/nsp4 junction is essential for DMV formation. (A) 293T cells were transfected with indicated plasmids and analyzed using Western blotting. nsp3 was detected with anti-SARS-nsp3 serum, and nsp4 was detected with anti-V5 monoclonal antibody. (B to E) HuH-7 cells were transfected with mutant nsp3-4 constructs individually (B and C) or cotransfected with the PL^pro^ domain of nsp3 (D and E) and analyzed using EM. Red arrows point at zippered ER, and in panel E, some putative DMVs are indicated with red asterisks. N, nucleus; M, mitochondria; LD, lipid droplet. Bars, 500 nm.

To gain more insight into the biogenesis of coronavirus DMVs, we set out to determine the role of the nsp3/nsp4 cleavage event. We surmised that the membrane modifications induced by an uncleaved nsp3-4 polyprotein could differ from those triggered by the (cleaved or coexpressed) nsp3 and nsp4 subunits. We transfected HuH-7 cells with plasmids encoding nsp3-4 carrying either a mutated nsp3/nsp4 cleavage site (GG>AA) or a catalytic site mutation in the nsp3 PL^pro^ domain (C1592A) that inactivates the protease (50). In both cases, only the uncleaved nsp3-4 precursor was observed (Fig. 5A). Interestingly, DMVs were no longer found and instead we detected concentric structures consisting of zippered ER that mostly lacked the pronounced curvature present in DMVs (Fig. 5B and C). Cotransfection of the cells with a plasmid encoding the active PL^pro^ domain restored the nsp3/nsp4 cleavage in the nsp3-4 C1592A mutant polyprotein but not in the nsp3-4 polyprotein with the mutated cleavage site (Fig. 5A). Accordingly, expression of PL^pro^ together with the nsp3-4 cleavage site mutant (Fig. 5D) did not alter the structures observed. In contrast, when transcleavage of the nsp3/nsp4 site, by coexpression of PL^pro^ with the nsp3-4 C1592A polyprotein, was achieved, DMV formation was at least partially restored and resulted in a mixture of abundant DMV and zippered ER profiles (Fig. 5E), as observed before (Fig. 2E and F). Expression of PL^pro^ by itself did not have a membrane-remodeling effect. These results clearly showed that the nsp3-4 precursor is able to induce the membrane pairing required to form zippered ER but that cleavage of the nsp3/nsp4 junction is essential for the formation of DMVs.

### SARS-CoV nsp3 and nsp4 are also sufficient to induce DMV formation.

Recently, Angelini et al. reported that, in the case of SARS-CoV, nsp3, nsp4, and nsp6 are all required for the formation of DMVs when these proteins are transiently expressed as individual subunits (42). In their two-dimensional (2D) imaging study, coexpression of SARS-CoV nsp3 and nsp4, in the absence of nsp6, led to the formation of so-called maze-like bodies (MLBs), large clusters of double-membrane structures that were interpreted as closely packed double-membrane tubules, not vesicles. Since our MERS-CoV tomography data (Fig. 3) established that DMV formation can be triggered just by coexpression of nsp3 and nsp4, the interpretation of Angelini et al. suggested that these subunits of MERS-CoV and SARS-CoV differ in their ability to induce DMV formation in the absence of nsp6.

**FIG 6:**
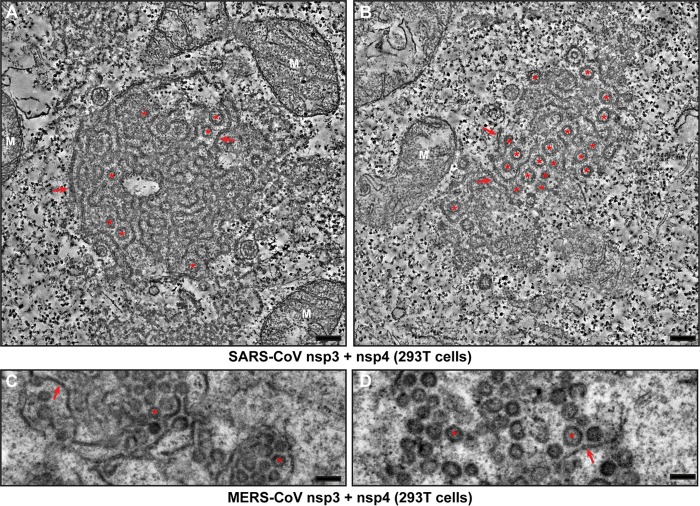
SARS-CoV nsp3 and nsp4 also suffice to induce DMV formation. (A and B) 293T cells were cotransfected with plasmids encoding SARS-CoV nsp3 and nsp4 and fixed for ET analysis 24 h posttransfection. Two virtual slices (8.5 nm) from reconstructed tomograms (available as Movies S3 and S4, respectively) are shown. Red arrows indicate zippered ER, and red asterisks indicate all the DMVs in this virtual slice that were fully reconstructed in the tomogram. (C and D) 293T cells were cotransfected with plasmids encoding MERS-CoV nsp3 and nsp4 and fixed for conventional EM analysis 24 h posttransfection. DMVs are indicated with red asterisks, and red arrows point at zippered ER. Bars, 250 nm.

To address this issue, we coexpressed nsp3 and nsp4 of either virus, using the same experimental setup previously used for SARS-CoV by Angelini et al. (293T cells transfected using lipofection), and employed ET for a comparative analysis in 3D. Coexpression of SARS-CoV nsp3 and nsp4 led to the formation of MLBs very similar to those observed by Angelini et al. (42), with areas of zippered ER, often clustered as regularly spaced profiles, and circular double-membrane profiles (Fig. 6A and B). The latter were postulated to be cross sections of double-walled tubules, of which the regularly spaced zippered ER profiles would then represent longitudinal sections (42). The fact that the spacing between clustered zippered ER profiles roughly coincided with the diameter of the circular profiles supported this interpretation; however, Angelini et al. also acknowledged that ET would be required for its validation. To determine whether the circular profiles in the MLBs represented tubular or vesicular structures, we now used ET to analyze several MLBs, two of which are shown in Fig. 6A and B. In one of those images, zippered ER is the dominant structure (Fig. 6A; Movie S3), whereas the other mainly contained circular double-membrane profiles (Fig. 6B; Movie S4). In both tomograms, we could detect multiple double-membrane profiles that increase and decrease in diameter when progressing through the tomogram and ultimately disappear (marked with green dots in the tomogram movies), indicating that they represent vesicles rather than tubules. In fact, no tubular structures were observed in the tomograms. The presumed longitudinal views of tubular structures turned out to consist of zippered ER winding through the MLB.

10.1128/mBio.01658-17.6 MOVIE S3  Three-dimensional reconstruction of the membrane structures induced by coexpression of SARS-CoV nsp3 and nsp4. Tomogram of the region shown in Fig. 6A, representative of areas rich in zippered ER that can be observed in 293T cells cotransfected with nsp3 and nsp4. Even in this type of region, in which zippered ER is predominant, some DMVs could be unambiguously identified. The movie shows consecutive virtual slices (1.67 nm thick) along the *z* axis of the 3D reconstruction and then loops back, marking with green dots all the DMVs that were fully reconstructed. In the tomogram, DMVs are recognizable as double-membrane circular profiles that decrease in size from the equator of the vesicle (largest size) until, if the DMV is fully contained in the section, they disappear. Bar, 500 nm. Download MOVIE S3, AVI file, 19.3 MB. Copyright © 2017 Oudshoorn et al. 2017 Oudshoorn et al. This content is distributed under the terms of the Creative Commons Attribution 4.0 International license.

10.1128/mBio.01658-17.7 MOVIE S4  Three-dimensional reconstruction of the membrane structures induced by coexpression of SARS-CoV nsp3 and nsp4. Tomogram of the region shown in Fig. 6B, typical of 293T cells cotransfected with SARS-CoV nsp3 and nsp4, containing abundant DMV. The movie progresses first along the *z* axis of the reconstruction through consecutive virtual slices (1.67 nm thick) and then loops back, marking with green dots all the DMVs that were fully contained in this section. Bar, 500 nm. Download MOVIE S4, AVI file, 19.6 MB. Copyright © 2017 Oudshoorn et al. 2017 Oudshoorn et al. This content is distributed under the terms of the Creative Commons Attribution 4.0 International license.

For MERS-CoV, coexpression of nsp3 and nsp4 in 293T cells led to the formation of numerous circular double-membrane profiles together with some zippered ER (Fig. 6C and D), which strongly resembled what we had observed in HuH-7 cells previously. Taken together, our ET results make it clear that, in the case of SARS-CoV as well, coexpression of nsp3 and nsp4 suffices for the induction of DMV formation and strongly suggest that this is a common feature among betacoronaviruses.

## DISCUSSION

The generation of membranous organelles that support their replication machinery is a universal mechanism among positive stranded RNA viruses infecting eukaryotes. The formation of these ROs is induced by viral proteins (32, 51, 52), which are largely uncharacterized in most instances, and appears to be also reliant on host factors, some of which have been identified as important players (53, 54). In this study, focusing on betacoronaviruses, we sought to identify the viral proteins required to induce the formation of DMVs, the most prominent membrane structure formed during coronavirus infection. Using ET, we found that coexpression of nsp3 and nsp4 of either SARS-CoV or MERS-CoV was required and sufficient to trigger the formation of ER-derived DMVs. Moreover, the 3D architecture of these membrane structures was similar to what has been observed during betacoronavirus infection (19). The DMVs formed upon coexpression of nsp3 and nsp4 were closed, with no detectable opening connecting the DMV interior and the surrounding cytosol, whereas their outer membrane generally was continuous with those of other DMVs and/or with (modified) ER. Our data importantly alter the conclusions of an earlier SARS-CoV study (42), which was based on the transient coexpression of SARS-CoV nsp3 and nsp4 from separate plasmids and the 2D imaging of the resulting membrane structures. The observation of maze-like bodies and circular double-membrane profiles, which were interpreted to represent tubular structures, led these authors to conclude that coexpression of SARS-CoV nsp3 and nsp4 was not sufficient for DMV formation. Using ET, we could now show that the circular profiles observed in these maze-like bodies are in fact DMVs (Fig. 6A and B), suggesting that the basic capability of nsp3 and nsp4 to induce DMV formation probably is a common feature of betacoronaviruses. These findings also highlight the importance of 3D analysis as a tool to ascertain and characterize the ultrastructure of membranous viral ROs.

Interestingly, in the case of the arterivirus equine arteritis virus (EAV), nsp2 and nsp3 were found to be required and sufficient for DMV formation (18, 41). At least to a certain extent, these proteins can be considered the functional equivalents of coronavirus nsp3 and nsp4, respectively, as they share a number of features like the presence of multiple membrane-spanning domains and a papain-like protease in the upstream protein that cleaves the junction between the two subunits. They also occupy comparable positions in the replicase polyproteins, suggesting that there may also be similarities in terms of the relative order in which these subunits are synthesized, released, and targeted to the membranes that they transform (17). These functional similarities and the potential to trigger DMV formation may thus be shared by these proteins of all coronaviruses and arteriviruses and possibly extend to other branches of the order *Nidovirales*, like the poorly studied ronivirus and mesonivirus families.

Our findings also shed more light on DMV biogenesis, for which two models have been proposed that are not mutually exclusive. The first has been termed “double budding,” where a vesicle would first bud into the ER lumen and then bud out again to acquire a second membrane. The alternative model is based on “wrapping”: membranes would first pair or “zipper” and then curve and finally form a closed DMV after a membrane fission event (18, 55). The frequent observation of zippered ER after coexpression of nsp3 and nsp4 and multiple EM images in which zippered ER seemed to wrap into a DMV (e.g., Fig. 3B) suggest that this structure is a DMV precursor. Interestingly, whereas the uncleaved nsp3-4 precursor was able to induce the pairing of ER membranes, DMV formation occurred only upon cleavage of the nsp3/nsp4 junction, which strongly suggests that membrane pairing is an early step in DMV formation. Our findings contrast with what was observed previously for arteriviruses, where cleavage of the nsp2/3 junction was not required for the formation of DMVs in an expression system (56). Together, our observations favor the wrapping model for DMV formation, and even though the existence in parallel of a double budding mechanism cannot be formally ruled out, the current data add to the mounting evidence pointing toward double-membrane wrapping as the central mechanism for DMV formation. For the distantly related arteriviruses (18, 57) and the unrelated picornaviruses (30, 31), putative wrapping intermediates have been also described, indicating that this might be a common mechanism of DMV biogenesis among positive stranded RNA viruses.

Several steps are required for DMV biogenesis: pairing of membranes, membrane curvature (both positive and negative), and fission (18, 55). In the wrapping model for DMV biogenesis, membrane pairing is an early step that may be mediated directly by interactions between the viral proteins inducing DMV formation. The interaction(s) between nsp3 and nsp4 that we described here for MERS-CoV may be sufficient to facilitate membrane pairing. Similar observations have been made for SARS-CoV and MHV (42, 48, 58). The most likely candidate regions for this kind of interactions are the luminal loops of nsp3 and nsp4 (33, 35) that are located in the TM1 and TM2 regions, respectively, as these could interact with their counterparts on the opposite side of the ER cisterna, thus inducing membrane pairing. This view is partly supported by studies on MHV and SARS-CoV for which a truncated nsp3 lacking the region upstream of TM1 coexpressed with nsp4 was sufficient to induce membrane pairing but not the formation of DMVs (48, 58). This suggests that, although the cytosolic N-terminal region of nsp3 is required for complete DMV formation, the TM1 region (together with nsp4) may be sufficient to induce membrane pairing.

In principle, the liberation (by PL^pro^-mediated cleavage of the nsp3/nsp4 junction) and presumed membrane insertion of the hydrophobic N-terminal domain of nsp4 may be an important determinant of the ultimate transmembrane configuration of this protein, potentially with direct implications for the transformation of zippered ER into DMVs. However, we found no major differences between DMVs induced by expression of self-cleaving MERS-CoV nsp3-4 and those induced by coexpression of nsp3 and nsp4, suggesting that nsp4 is properly inserted in the membrane when individually expressed. The concentric zippered ER observed after expression of the uncleaved nsp3-4 polyprotein could then reflect an intermediate stage in which the lack of nsp3/nsp4 cleavage prevents proper membrane remodeling. The proximal (and largest) luminal loop of nsp4 contains an N-linked glycosylation site (N2985 in MERS-CoV), similar to the glycosylation site(s) in SARS-CoV and MHV (34, 37, 40). Analysis of the use of that site in proteolytically processed nsp4 and the uncleaved nsp3-4 precursor could provide insight into the sequence of events leading to the membrane insertion of MERS-CoV nsp4.

Although our data establish that expression of nsp3 and nsp4 suffices for coronaviral DMV formation, the precise role in this process—if any—of the nsp6 transmembrane subunit remains unclear. Our MERS-CoV data suggest that, compared to cells expressing nsp3 and nsp4 only, coexpression of cleaved nsp6 does not affect DMV formation, nor does it lead to the formation of additional structures like CM or spherules. However, coexpression of SARS-CoV nsp3, nsp4, and nsp6 was previously reported to induce CM formation in addition to DMVs (42). Additionally, expression of nsp6 alone resulted in the formation of small single-membrane vesicles near the microtubule organizing center (42), which suggested that nsp6 may have membrane proliferation and vesiculation abilities that could play a role in RO formation.

When MERS-CoV nsp6 was retained in an unprocessed nsp4-6 precursor, DMVs were no longer formed and membrane clusters that resemble cubic membranes appeared (Fig. 4C). It has been proposed that the CM formed by coronaviruses are in fact a form of cubic membranes (59). This might be related to observations that, compared to DMVs, CM are mostly formed relatively late in infection (19, 21, 24) when viral proteins or polyprotein fragments accumulate. It is conceivable that such accumulation could lead to aggregation, misfolding, and/or impaired polyprotein processing resulting in the formation of cubic membranes. In other words, there could be a link between the status of polyprotein processing in the nsp4-6 region and the membrane structures formed. Along the same lines, the observation that blocking the cleavage at the nsp3/4 junction impedes DMV formation (see above) directly implicates polyprotein processing in the control of membrane remodeling, possibly by facilitating conformational changes required for specific interactions with the membrane and/or between nsp3 and nsp4. For an unrelated DMV-forming virus, hepatitis C virus (HCV), it was recently shown that DMV formation became less efficient when the proteolytic cleavage of the NS4B/5A site in the viral polyprotein was accelerated, which similarly suggests a role for the polyprotein processing in DMV formation (60).

The existence of different proteolytic processing intermediates containing nsp5 is well documented for arterivirus infection (61), although the role of the different precursors in DMV formation has not been studied in depth so far. Unfortunately, the kinetics of polyprotein processing by M^pro^ in MERS-CoV and SARS-CoV are still largely unknown. M^pro^’s enzymatic activity has mainly been assessed using recombinant nsp5 and peptide substrates *in vitro* (9, 11, 13, 50), but an analysis of the kinetics in a large (or larger) polyprotein setting is mostly lacking. Most information on the processing of the coronavirus nsp4-to-nsp10 region is derived from studies on other coronaviruses, such as MHV, IBV, and human coronavirus 229E (HCoV-229E) (the latter two being a gamma- and an alphacoronavirus, respectively) (9, 62, 63). An in-depth analysis of the kinetics of polyprotein maturation during coronavirus infection, the identification of nsp6-containing processing intermediates, and the investigation of their possibly distinct roles in membrane remodeling could help to further unravel the mechanisms underlying the formation of the coronavirus ROs.

## MATERIALS AND METHODS

### Cells, viruses, and antibodies.

HuH-7 cells (kindly provided by Ralf Bartenschlager, Heidelberg University) were grown in Dulbecco’s modified Eagle’s medium (DMEM; Lonza) supplemented with 8% (vol/vol) fetal calf serum (FCS; Bodinco), 2 mM l-glutamine (PAA Laboratories), and nonessential amino acids (PAA Laboratories). 293T cells (kindly provided by the Virgin lab, Washington University School of Medicine in St. Louis, MO) were cultured in DMEM with 10% (vol/vol) FCS. All cell culture media contained 100 U/ml penicillin and 100 µg/ml streptomycin. Infection of HuH-7 cells with MERS-CoV (EMC/2012 strain kindly provided by Ron Fouchier, Erasmus Medical Center, The Netherlands [3, 4]) was performed as previously described (21).

Primary antibodies used were mouse anti-HA (clone HA.C5; Abcam), mouse anti-β-actin (clone AC-74; Sigma), and mouse anti-V5 (clone 2F11F7; Thermo Fisher). A rabbit serum recognizing SARS-CoV-nsp3 that cross-reacts with MERS-CoV nsp3 has been previously described (21, 22). A polyclonal rabbit serum was used against a combination of two MERS-CoV nsp5 peptides, SGLVKMSHPSGDVEAC (amino acids 3248 to 3263 of pp1a) and CPADQLSDPNYDALLI (amino acids 3291 to 3306), which was produced by Eurogentec.

### Plasmid construction and transfection.

Human codon-optimized coding sequences of MERS-CoV nsp3-6 were designed using GeneArt, ordered from Thermo Fisher in four fragments, and subsequently assembled in low-copy-number vector pACNR1180 (64) using conventional cloning. The precise parts of MERS-CoV pp1a used for polyprotein constructs are outlined in Table S1 in the supplemental material. The nsp4 construct included the 21 C-terminal aa of nsp3 to prevent the N-terminal hydrophobic region of nsp4 from acting as a signal sequence, which could result in improper membrane insertion. In all constructs with a C-terminal myc or V5 tag, the C-terminal glutamine of the viral sequence was omitted to prevent the removal of the tag by M^pro^. The SARS-CoV nsp3 gene (Frankfurt 1 strain, pp1a amino acids 819 to 2740) was synthesized by Bio Basic Inc. (Ontario, Canada). Coding sequences were transferred to the pCAGGS expression vector (Addgene) for expression. pCAGGS-SARS-nsp4 was described previously (42). 293T cells were transfected using Lipofectamine 2000 (Thermo Fisher) according to the manufacturer’s instructions. HuH-7 cells were transfected using a Nucleofector 2b device (Lonza) with Nucleofector kit T (Lonza) in 6 × 10^6^ cells and 12 µg of plasmid DNA per transfection. Cotransfections were carried out with equimolar amounts of plasmids.

### Western blotting.

Cells were lysed in 2× Laemmli sample buffer (50 mM Tris-HCl, pH 6.8, 20% [vol/vol] glycerol, 4% [wt/vol] sodium dodecyl sulfate [SDS], 20 mM dithiothreitol, 0.02 mg/ml bromophenol blue) and separated by electrophoresis on SDS-polyacrylamide gels. Proteins were transferred to polyvinylidene fluoride membranes (Amersham) using a Trans-Blot Turbo transfer system (Bio-Rad). Blots were blocked with 5% (wt/vol) ELK skimmed milk powder (Campina) in phosphate-buffered saline (PBS) supplemented with 0.05% (vol/vol) Tween 20. Secondary horseradish peroxidase (HRP)-conjugated antibodies (Dako) and ECL Plus Western blotting substrate (Thermo Fisher) were used to visualize protein signal.

### Immunofluorescence microscopy.

After electroporation, HuH-7 cells were seeded on coverslips and fixed 24 h later with 3% (wt/vol) paraformaldehyde in PBS. Samples were permeabilized with 0.2% (vol/vol) Triton X-100 and incubated with antibodies, including fluorescent conjugates, diluted in 5% (wt/vol) bovine serum albumin (BSA) in PBS. Nuclei were stained with 1 µg/ml Hoechst 33258. After embedding with Prolong Gold (Thermo Fisher), samples were analyzed with a Leica TCS SP8 confocal laser scanning microscope, which was equipped with a 63× objective (numerical aperture [NA] 1.40; 1 Airy unit) and a Leica HyD hybrid detector.

### Metabolic labeling and IP.

293T cells were metabolically labeled with 100 µCi/ml [^35^S]methionine and [^35^S]cysteine (EXPRE^35^S^35^S protein labeling mix; PerkinElmer) from 4 h posttransfection onward. Cells were lysed at 18 h posttransfection in 20 mM Tris-HCl (pH 7.6), 150 mM NaCl, 0.5% (wt/vol) deoxycholic acid, 1% (vol/vol) Nonidet P-40, and 0.1% (wt/vol) SDS. Lysates were diluted in immunoprecipitation (IP) buffer (20 mM Tris-HCl [pH 7.6], 150 mM NaCl, 5 mM EDTA, 0.1% [wt/vol] deoxycholate [DOC], 0.5% [vol/vol] NP-40, and 0.5% [wt/vol] SDS) and incubated with antibody overnight. Antibody-protein complexes were then pulled down using protein A and protein G Sepharose beads (GE Healthcare), which were first blocked with 2% (wt/vol) BSA in PBS, and incubated for several hours. After repeated washing of the beads with IP buffer, proteins were eluted by heating in 2× Laemmli sample buffer. After separation on large 10% polyacrylamide gels and gel drying, signal was visualized using an Imaging Screen-K (Bio-Rad) and a Typhoon 9410 scanner (GE Healthcare).

### Electron microscopy.

Transfected HuH-7 or 293T cells were fixed 24 h posttransfection in 1.5% (wt/vol) glutaraldehyde in 0.10 M cacodylate buffer (pH 7.4) for 1 h at room temperature. After washing in 0.14 M cacodylate buffer, samples were postfixed and stained at 4°C with 1% (wt/vol) osmium tetroxide in 0.10 M cacodylate buffer for 1 h. After washing with 0.14 M cacodylate and Milli-Q water, cells were scraped and stained with 1% (wt/vol) tannic acid in Milli-Q water on a 3D rotator for 1 h at room temperature. Following washing with Milli-Q water, cells were spun down in heated 3% (wt/vol) agar in PBS, and after solidification, pellets were excised, cut into small blocks, and dehydrated in increasing concentrations of ethanol. Samples were embedded in epoxy resin (LX-112; Ladd Research), and after polymerization, 100-nm sections were placed on mesh-100 copper EM grids covered with a carbon-coated Pioloform layer. Following poststaining with 7% (wt/vol) uranyl acetate and Reynolds lead citrate, samples were analyzed on an FEI Tecnai 12 BioTwin microscope equipped with an Eagle cooled slow-scan charge-coupled device (CCD) camera (FEI) and operated at 120 kV. Measurements of circular profiles from 2D EM images were done with ImageJ software and Aperio Imagescope software (Leica). Circular profiles were measured over their longest and shortest axes, and the geometric mean of those values was used as the diameter. One hundred circular profiles were measured for each condition.

### Electron tomography.

Sections of 150-nm thickness were cut from the resin-embedded blocks of transfected HuH-7 or 293T cells prepared as described above. Prior to poststaining, colloidal gold particles of 10 nm were applied to both sides of the EM grid to serve later as fiducial markers for alignment. Tomography data were recorded on an Eagle CCD camera (FEI) in an FEI Tecnai 12 BioTwin (HuH-7 samples) or a Twin (293T samples) electron microscope operated at 120 kV, with the grids mounted on a 2040 Fischione tomography holder. Dual-axis tilt series of the regions of interest were collected using Xplore3D software (FEI) at magnifications that resulted in a pixel size of 1.7 nm (BioTwin data) or 1.4 nm (Twin data). The angular coverage for each single-axis tilt series was 130° sampled in increments of 1°. Alignment of the tilt series and tomogram reconstruction by weighted back-projection were performed in IMOD (65).
