# Supplementary figures and images for: Expression and Cleavage of Middle East Respiratory Syndrome Coronavirus nsp3-4 Polyprotein Induce the Formation of Double-Membrane Vesicles That Mimic Those Associated with Coronaviral RNA Replication
Source: mBio. 2017 Nov 21;8(6):e01658-17. doi: 10.1128/mBio.01658-17 (PMC5698553; doi:10.1128/mBio.01658-17)

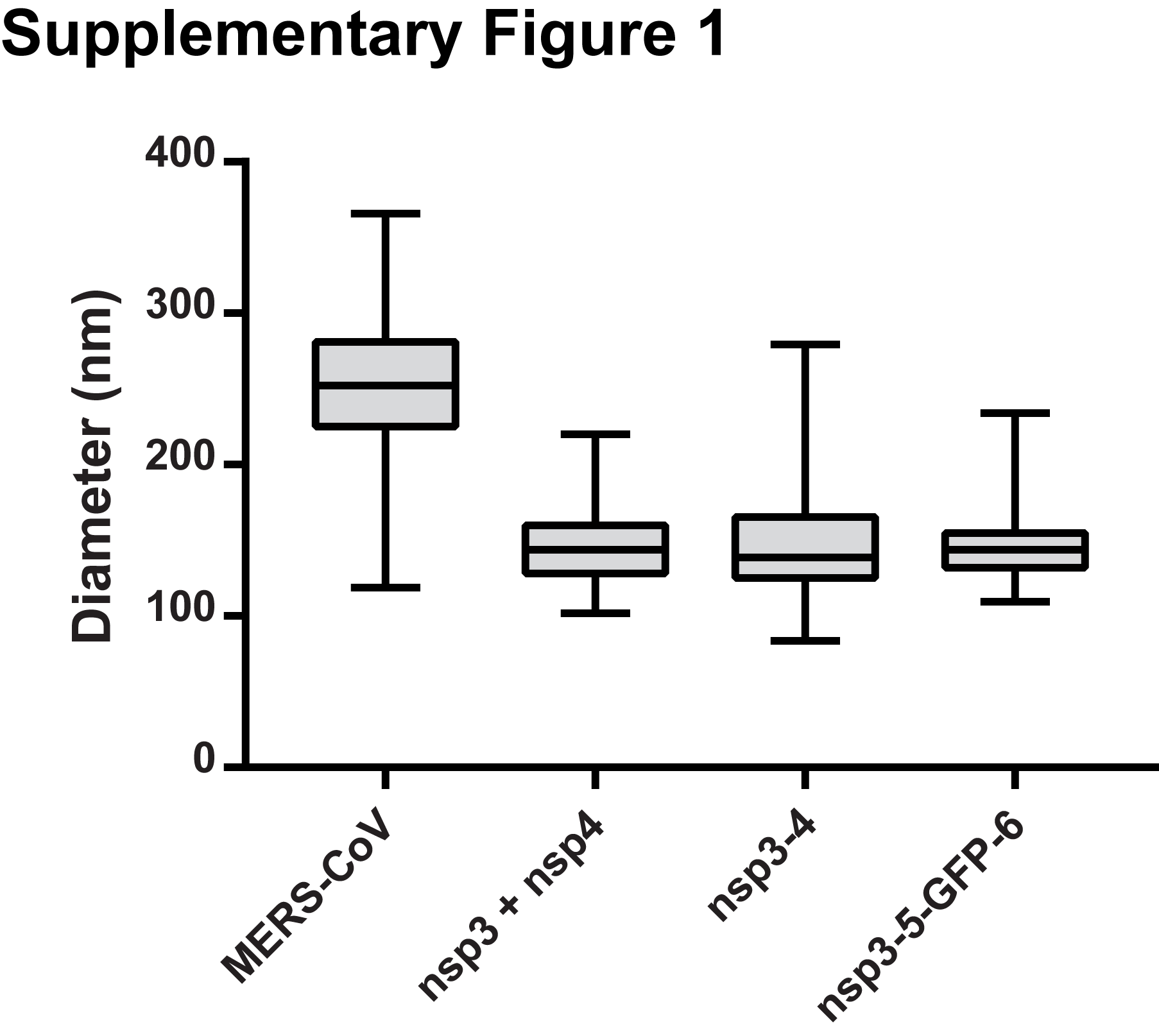

Supplement: FIG S1 [file mbo006173595sf1.tif]

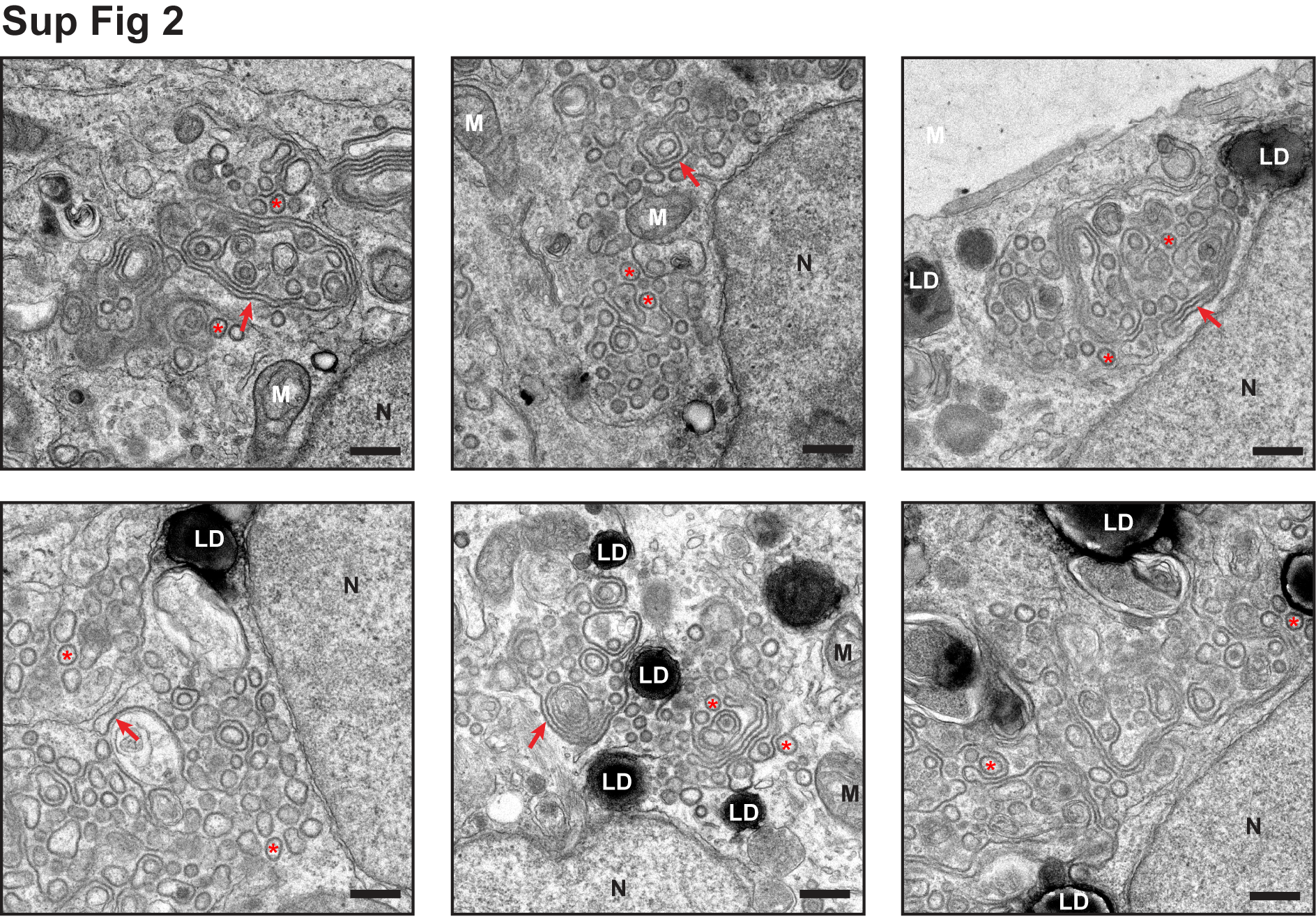

Supplement: FIG S2 [file mbo006173595sf2.tif]
